# Zellweger syndrome; identification of mutations in *PEX19* and *PEX26* gene in Saudi families

**DOI:** 10.1080/07853890.2024.2447400

**Published:** 2025-01-06

**Authors:** Abdulfatah M. Alayoubi, Ambreen Ijaz, Abdul Wali, Jamil A. Hashmi, Azizah Alharbi, Sulman Basit

**Affiliations:** a Department of Basic Medical Sciences, College of Medicine & Center for Genetics and Inherited Diseases, Taibah University Medina, Medina, Saudi Arabia; b Department of Zoology, Sardar Bahadur Khan Women’s University Quetta, Quetta, Pakistan; c Department of Biotechnology, Faculty of Life Sciences & Informatics, BUITEMS, Quetta, Pakistan; d Department of Pediatrics, Medina Maternity and Children Hospital, King Salman bin Abdul Aziz Medical City, Medina, Saudi Arabia

**Keywords:** Peroxisome biogenesis disorders, severe Zellweger spectrum disorder, whole exome sequencing, *PEX19* gene, *PEX26* gene

## Abstract

**Background:**

Peroxisome biogenesis disorders (PBD) affect multiple organ systems. It is characterized by neurological dysfunction, hypotonia, ocular anomalies, craniofacial abnormalities, and absence of peroxisomes in fibroblasts. PBDs are associated with mutations in any of fourteen different *PEX* genes, which are involved in peroxisome biogenesis. Zellweger spectrum disorder (ZSD) is a severe form of PBD. More than 90% of the ZSD cases have mutations in *PEX1*, *PEX6*, *PEX10*, *PEX12*, and *PEX26*. Mutations in the PEX19 gene are rarely associated with PBD/ZSD; however, a large proportion of *PEX26* mutations are associated with ZSD.

**Methods:**

We recruited two Saudi families with multiple affected individuals with dysmorphic features, including hypertelorism, large open fontanelles, generalized hypotonia, and epicanthal folds with poor reflexes since birth. Whole exome sequencing (WES) and Sanger sequencing was performed to identify the genetic cause. The frequency and pathogenicity of the identified mutations were assessed using various online bioinformatics tools.

**Results:**

WES identified a novel nonsense variant (c.367C > T) in the *PEX19* gene in family A patients. This nonsense mutation was predicted to cause premature termination (p.Gln123*). A previously reported synonymous variant (c.228C > T; p.Gly76Gly) in *PEX26* was found in a patient from family B. Both variants were segregating in an autosomal recessive manner in the respective families.

**Conclusion:**

The present study has added a novel nonsense mutation to the mutation spectrum of *PEX19*, which is the second null mutation identified to date. Moreover, in this study, the importance of a synonymous exonic variant of *PEX26* close to the splice donor site was explored in relation to pre-mRNA splicing and resulting disease manifestations.

## Introduction

Peroxisomes are subcellular ubiquitous membranous organelles found exclusively in eukaryotic cells. Their number in the cells depends on the cellular metabolism and physiological circumstances of the cells, and they are most abundantly present in the liver and kidney tubular cells [1]. They are provided with a rich granular matrix with hundreds of enzymes that catalyze various metabolic pathways, such as β-oxidation of very long chain fatty acids (VLCFA), prostaglandins, and leukotrienes, biosynthesis of ether lipids such as plasmalogens, and detoxification of reactive oxygen species as well as xenobiotics [2–4]. β-oxidation of fatty acids occurs both in mitochondria and peroxisomes through the same pathways; however, mitochondria oxidize short-, medium-, and long-chain fatty acids, but peroxisomes preferentially catalyze very long-chain fatty acids by β-oxidation and branched chain fatty acids (methyl branched phytanic acid) by α-oxidation [5,6]. They also generate and subsequently inactivate reactive oxygen species during some metabolic reactions [3]. Peroxisomes are devoid of DNA, so all the proteins required for the assembly and functions of peroxisomes are encoded by nuclear genes and synthesized in the cytosol on free polyribosomes, after which they migrate to peroxisomes [7]. Peroxisomes have the ability to modulate their number in cells in response to metabolic requirements and are formed from pre-existing organelles as well as derived *de novo* from the endoplasmic reticulum [8–11].

Proteomics, bioinformatics, and genetic studies have identified 85 different peroxisomal structural and functional proteins in humans [12]. The biosynthesis of peroxisomes is harnessed by the coordinated action of different proteins called peroxins or PEX, which are crucial for the proliferation of peroxisomes, formation of new peroxisomal membranes, and synthesis and import of matrix enzymes from the cytosol [13–15]. Mutations in any of these genes encoding peroxin proteins result in a heterogeneous group of autosomal recessive genetic disorders called peroxisomal biogenesis disorders characterized by severe hypotonia, large anterior fontanel, poor neonatal reflexes, epicanthal folds, prominent forehead, metopic sutures, high arched palate, broad nasal bridge, loss of hearing, anteverted nares, and ocular abnormalities [7,16–18]. PBDs are divided into two main types: rhizomelic chondrodysplasia punctate (RCDP) and sever Zellweger spectrum disorders (ZSDs) [19]. RCDP is clinically characterized by facial dysmorphism, microcephaly, rhizomelia (proximal shortening of limbs), cataracts, small stature, psychomotor retardation, and calcifications in the cartilage with metaphyseal abnormalities [20,21]. The primary features of ZSDs include liver dysfunction, neurological abnormalities leading to developmental delays, adrenocortical dysfunction accompanied by vision impairment, and hearing impairment [22,23]. The traditional classification of ZSDs, based on the severity of clinical symptoms, includes Zellweger syndrome (ZS), neonatal adrenoleukodystrophy (NALD), and infantile Refsum disease (IRD), which refer to severe, intermediate, and mild forms, respectively. Patients with severe ZS experience profound neurological impairment, renal cysts, hepatic dysfunction, elevation of liver function enzymes, and polymicrogyria with frequent multisystem involvement. Patients with severe ZS do not survive beyond the age of two years whereas those in the intermediate and milder categories are often recognized later in childhood and develop progressive clinical symptoms over time [7,16–18]. Approximately 80% of all PBD patients belong to the ZS category, with an incidence of 1 per 50000 births in the US and 1 per 500,000 births in Japan [24–26].

Fourteen complementation groups (CGs) of PBDs have been identified to date. The CGs and associated genes are listed in Table 1. To date, mutations in 14 PEX genes have been reported for PBDs in which the genes *PEX* 1, 2, 5-7, 10, 11β, 12-14 and 26 are essential for the import of matrix enzymes from the cytosol into peroxisomes, and *PEX* 3, 16, and 19 work for the biogenesis of peroxisomes and assembly of peroxisomal membranes [7,27–29]. More than 90% of ZSD cases have mutations in *PEX1, PEX6, PEX10, PEX12* and *PEX26*, of which *PEX1* and *PEX26* mutations contribute to 70% and 10%, respectively [26]. *PEX19* mutations have been reported in developmental disorders, PBDs, ZS, and progressive myoclonic epilepsy (Table 2). Mutations in *PEX19* gene are one of the least common causes of ZS disorders [7] and only a few ZS cases with *PEX19* mutations have been reported in the literature. Pathogenic mutations in *PEX3, PEX16* and *PEX19* cause affected cells to be devoid of peroxisomes [36,38–40]. ZS patients with *PEX26* mutations have seriously impaired peroxisomal matrix protein import with loss of protein levels and related functions, resulting in severe clinical symptoms in patients [11,41,42].

**Table 1.** Complementation groups (CG), PEX genes and their chromosomal locations.

Gene	Chromosome Location	Complementation Group
*PEX1*	7q21-22	1
*PEX2*	8q21.1	10
*PEX3*	6q23-24	12
*PEX5*	12p13.3	2
*PEX6*	6p21.1	4(6)
*PEX7*	6q21-22.2	11
*PEX10*	1p36.32	7(5)
*PEX12*	17q21.1	3
*PEX13*	2p14-16	13
*PEX14*	1p36.22	15
*PEX16*	11p11.11	9
*PEX19*	1q22	14
*PEX26*	22q11.21	8
*PEX1*	7q21-22	1
*PEX2*	8q21.1	10
*PEX3*	6q23-24	12
*PEX5*	12p13.3	2

**Table 2.** List of all the identified mutations in *PEX19* (from HGMD professional).

Codon Change	HGVS	Amino Acid Change	Reported phenotype	References
TCG-TTG	c.161C > T	Ser54Leu	Peroxisomal biogenesis disorder	[30]
GCG-GTG	c.254C > T	Ala85Val	Progressive myoclonic epilepsy	[31]
GAT-AAT	c.352G > A	Asp118Asn	Developmental disorder	[32]
CAG-TAG	c.769C > T	Gln257Term	Zellweger syndrome, complementation group J	[33]
CAA-TAA	c.367C > T	p. Glu123Term	Zellweger syndrome	Present study
CTC-TTC	c.847C > T	Leu283Phe	Developmental disorder	[34]
CAGTTC^**^106^**CAAAaGCTCTCAGAG	c.320delA	p.Lys320fsX13	Peroxisomal biogenesis disorder	[35]
CTGGAT^**^254^**CTTAaTGCAGCAG_EI_GT	c.763_764insA	p.Met255AsnfsX2	Zellweger syndrome, complementation group J	[36]
UUG-UAG	c.281T > A	Leu94Term	Zellweger syndrome	[37]

The gene *PEX19* consists of eight exons, located on chromosomal region 1q22 and spans a 9 kb region [43]. PEX19 is a hydrophilic acidic protein found predominantly in the cytoplasm and is associated with the peroxisome membrane by farnesylation of its C-terminal region after binding with pex3 [44,45]. It has unique N- and C-terminal domains consisting of amino acids 1–44 and amino acids 160-300, respectively [46–48]. The N-terminal region is highly flexible and capable of forming alpha helices, whereas the C-terminal region contains a CAAX-box that harbors the property of post-translational farnesylation [11,36]. Farnesylation causes structural rearrangements in the C-terminal globular domain, which functions as an allosteric regulator of PEX19 binding peroxisomal membrane proteins (PMPs) [49,50].

*PEX26* gene is located on chromosome 22q11 (OMIM# 608666) and encodes a 34-kDa peroxisome assembly factor called peroxin 26, comprising 305 amino acids that function in the localization of PEX1-PEX6 complexes to the peroxisomal membrane and are required for the import of peroxisome matrix proteins [7,51–53]. The C-terminal part has a transmembrane domain (amino acids 252-269) which is exposed to the matrix side, whereas the N-terminal part faces the cytosol with a PEX6 binding domain (amino acids 29-174) [52,54,55]. Homozygous pathogenic variants of *PEX26* have been reported to cause variable clinical symptoms, ranging from severe ZS to less severe IRD. Mutations near the N terminal are more deleterious to the structure and function of peroxisomes. The observed mutations in *PEX26* are spread across the gene; however, 70% of the detrimental mutations, including nonsense, splicing, and frameshift mutations, cause truncation of the N-terminal domain and its malfunction [56].

We recruited two consanguineous Saudi Arabian families (A and B). The affected neonates exhibited serious clinical manifestations of dysmorphism with generalized hypotonia after birth and were clinically diagnosed with connective tissue disorder (cardio-facio cutaneous syndrome) and acrocallosal syndrome. They were subjected to metabolic screening, followed by a necessary medical laboratory tests, and were referred for genetic testing. Mutation analysis by whole-exome sequencing identified a homozygous novel nonsense mutation in the *PEX19* gene in a patient from family A and a previously reported variant in *PEX26* gene in a patient from family B.

## Materials and methods

### Approval of the study and patients recruitment

**Figure 1.**
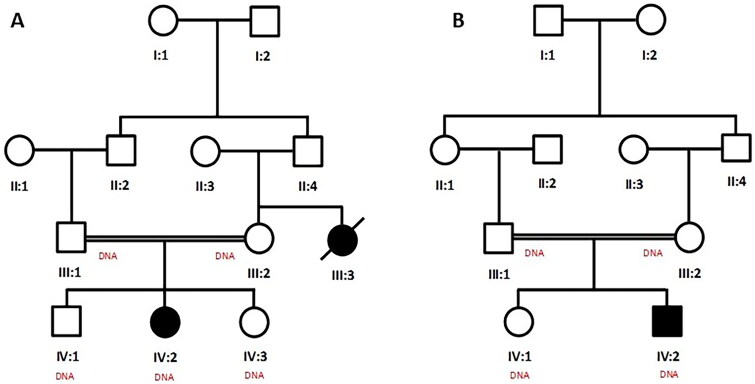
Pedigrees of consanguineous family A and B with Zellweger syndrome. Affected and unaffected individuals are represented by filled and unfilled symbols, respectively. Double line indicate consanguineous unions. Symbols with ‘DNA’ represent the samples available for the study.

This study was approved by the Institutional Review Board of the College of Medicine, Taibah University Medina, KSA. Patients from families A and B were examined at the Medina Maternity and Children Hospital (MMCH), KSA. The parents were first-degree cousins and did not have any genetic or metabolic disorders or any history of such disorders. Pedigrees were drawn based on information from adult members of the families (Figure 1). Written informed consent for participation in the study has been obtained from parents of all minor affected individuals. The infants were subjected to detailed clinical examination by a pediatric neurologist and neuro-radiologist at MMCH, KSA, followed by necessary laboratory tests, including MRI, ECHO, ultrasound, EEG, CT scan, LFT, and metabolic screening of blood and urine samples.

### Molecular analysis

#### DNA isolation and whole exome sequencing

Genomic DNA was isolated from peripheral blood samples using the QIAmp Blood Midi Kit (Qiagen) following standard protocols and quantified using a Nanodrop-1000 spectrophotometer (Thermo Fisher Scientific, Wilmington, and Massachuttes, USA).

WES was performed on DNA samples from patient (IV-I) from family A and patient (IV-1) from family B. The samples were enzymatically fragmented for whole-exome library preparation. All exonic regions of the genome comprising approximately 22000 genes were captured using xGen Exom Research Panelv2 (Integrated DNA Technologies, Coralville, Iowa, USA). Sequencing of the captured exonic regions was performed using a Novaseq 6000 (Illumina, San Diego, CA, USA).

#### Variant prioritization and bioinformatics analysis

Paired-end reads were processed and aligned to the reference human genome sequence GRCh37/hg19. Variant calling of single nucleotide polymorphisms (SNPs) and indels was conducted using in-house software and bioinformatic tools. All variants were prioritized based on patient phenotype and standard ACMG guidelines using VariantStudio software (Richards et al. 58). The steps followed were variant filtration, classification, and similarity scoring considering patient phenotypes.

Allele frequency was determined using an online population genome database, gnomAD (http://gnomad.broadinstitute.org/). All variants with minor allele frequency >0.01 in different public databases such as Exome Aggregation Consortium (ExAC), database of single nucleotide polymorphisms (dbSNP), Genome Aggregation Database (gnomAD), 1000 Genomes Project, and Exome Sequencing Project 6500 (ESP6500) were filtered out. Common variants not meeting the BA1 criterion of the ACMG guidelines were also filtered out [57]. The homozygous and biallelic variants of the genes associated with the disease phenotype in the literature and inheritance patterns in families were selected for further analysis. The pathogenicity of these variants was checked using scientific disease databases such as UniProt (https://www.uniprot.org/), ClinVar (https://www.ncbi.nlm.nih.gov/clinvar/) and HGMD database (professional), followed by verification of the pathogenicity of the data based on ACMG guidelines [57]. The pathogenicity of the mutation was also assessed using MutationTaster. Candidate variants were also analyzed by medical geneticists and physicians to determine their association with disease phenotypes.

#### Validation of variants by Sanger sequencing

The identified candidate variants were confirmed by Sanger sequencing. Genomic regions surrounding the variants were PCR amplified by forward and reverse primers (primers available on request) in all available samples of both family members. The PCR products were sequenced using an AB3500 genetic analyzer (Applied Biosystems, CA). The BIOEDIT sequence alignment software was used to analyze the sequencing results (http://www.mbio.ncsu.edu/BioEdit/bioedit.html).

## Results

### Clinical characteristics of patients

#### Patient from family A

The enrolled Saudi family (A) had an affected female infant (IV-1). The baby was delivered by emergency caesarean section with Apgar scores of 5 and 6 at 1 and 5 min, respectively. Antenatal scan showed polyhydramnios, kidney agenesis (single kidney), and ventriculomegaly. The patient experienced poor breathing and bradycardia immediately after birth. A detailed physical examination showed abnormal facial features with low-set ears, prominent premaxilla, and nose with a broad depressed nasal bridge. She had dysmorphic features, such as hypertelorism, increased skin folds, prominent calcaneus, joint laxity, open tented mouth, and periorbital puffiness. The proband had generalized hypotonia with poor reflexes and open anterior fontanelle. She had normal GIT and respiratory functions. Metabolic profile including very long chain fatty acids (VLCFA) were unremarkable. ECHO showed an abnormal septal defect and patent ductus arteriosus as signs of developmental disability. Based on these clinical features, the patient was initially diagnosed with connective tissue disorder/cardio-facio-cutaneous syndrome.

#### Patient from family B

A male patient (IV-1) from a consanguineous Saudi family (family B) was examined at the 14 days of age. He had an emergency delivery due to fetal distress, with good Apgar scores. Her antenatal scan revealed ventriculomegaly, and postnatal examination revealed dysmorphic features, including hypertelorism, prominent epicanthal fold, scaphocephaly, retrognathia, and sparse eyebrows. He had generalized hypotonia with poor reflexes, clinodactyly, an open anterior fontanelle, and bilateral clubfeet with overlapping toes. Neurological examination showed central hypotonia with an absence of the corpus callosum, asymmetrical supratentorial ventricular dilation (right lateral ventricle dilation), and multiple cysts in the caudothalamic area. Metabolic screening of blood and urine samples showed a normal range of metabolites, however, VLCFA profile was slightly elevated.

#### Molecular findings

WES identified a homozygous nonsense variant (c.367C > T; p. Gln123*) in exon 4 of *PEX19* in a female patient (IV-1) from family A and a homozygous synonymous variant (c.228C > T; p.Gly76Gly) in exon 2 of *PEX26* in a patient (IV-1) from family B. No other potentially candidate variant in *PEX* genes or any other gene was identified in the DNA of both patients.

**Figure 2.**
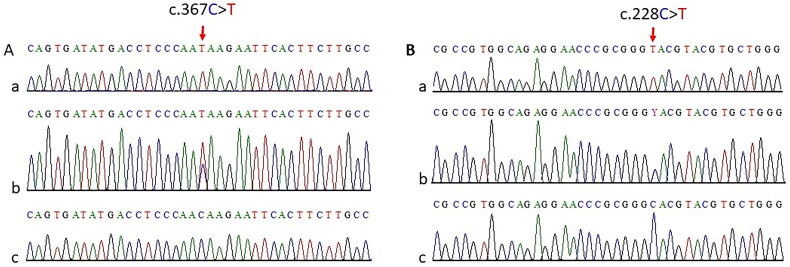
Partial sequence chromatogram of *PEX19* (A) and *PEX26* (B) gene flanking variants (c.367C > T and c.228C > T) identified in family A and B. The upper panels (a) represent the homozygous mutant sequence, the Middle panels (b) represent the heterozygous carrier and the lower panel (c) represent the wild type sequence. Arrows indicate position of the mutation.

The genomic region surrounding the respective variant sites was PCR-amplified in all available samples from both families and sequenced using Sanger sequencing. Alignment of Sanger sequencing data with reference genomic sequences revealed that the variants were present in a homozygous state in patients and in a heterozygous state in both parents, confirming an autosomal recessive pattern of inheritance (Figure 2). The mutation c.367C > T of *PEX19* gene results in the conversion of the glutamine (CAA) to stop codon (UAA) at position 123 and would result in premature termination of the encoded mRNA transcript, which is predicted to face the NMD pathway of the cells. The identified mutation has not been reported in any of the public databases, is categorized as pathogenic according to the ACMG guidelines, and is classified as damaging by SIFT, PolyPhen2, and MutationTaster, with an average CADD score of 40 (https://cadd.gs.washington.edu/). The variant c.228C > T is positioned near the splice donor site (GU) of exon 2 of *PEX26* and is predicted to affect splicing at pre mRNA level. This variant has been reported at an extremely low frequency in the gnomAD database. Moreover, the variant (c.228C > T) has been reported to be pathogenic [58].

## Discussion

Proxisomes are indispensable for the proper functioning of human organs because of their vital roles in anabolic and catabolic reactions. Their catabolic activity comprise α- and β-oxidation of very long chain fatty acids (VLCFAs), phytanic acids and pristanic acids. They also provide a home for the biosynthesis of cholesterol, bile acids, plasmalogens, and polyunsaturated fatty acids (FAs) as their anabolic functions [59–62]. A smooth working phase of peroxisomes in cells is maintained by independent mechanism of peroxisome biogenesis consisting of a series of steps comprising the import of matrix proteins, formation of peroxisomal membranes, peroxisomal proliferation, and growth [63]. Defects at any stage of peroxisome biogenesis impair its metabolic function, resulting in elevated levels of VLCFAs, cellular reactive oxygen species, and the accumulation of ether lipid precursors of plasmalogens [11,49,50]. Accordingly, partial or complete peroxisome dysfunction has been associated with inherited neuropathology, cancer, heart disease, obesity, and diabetes [64–66].

PBD is a heterogeneous group of autosomal recessive metabolic disorders caused by defects in peroxisomal biogenesis. The PEX proteins encoded by 14 different genes are involved in the biogenesis, homeostasis, and proliferation of peroxisomes [7]. Defects or mutations in any of the PEX genes are expressed as the variable clinical symptoms of PBD. Patients suspected of having PBD are diagnosed on the basis of clinical features, MRI findings showing polymicrogyria, pachygyria, delayed myelination, cortical malformation, and biochemical analysis suggesting abnormally elevated VLCFAs levels. They are further subjected to genetic screening to identify the disease-causing mutations. Owing to the genetic heterogeneity of PBD, next-generation sequencing (WES) is preferred for exact diagnosis. ZS is the most severe form of PBD, manifested by the complete absence of functional peroxisomes, resulting in a multisystem involvement. ZS clinical symptoms include hypotonia, seizures, craniofacial dysmorphism with high forehead, high arched palate, micrognathia, peculiar facial appearance, hydrocephalus, cardiac anomaly, genital abnormalities, liver dysfunction, generalized brain atrophy, cardiovascular malformations and pulmonary hypoplasia with early neonatal death [37]. ZS disorder, also known as cerebrohepatorenal syndrome, as major organs affected are kidneys, liver, and brain [67].

The generalized clinical characteristics of patients, in this study, included dysmorphic features, hypertelorism, pronounced epicanthal folds, scaphocephaly, hypotonia accompanied by ventriculomegaly, open anterior fontanelle, and neurodevelopmental delay. These observations are in accordance with the published clinical descriptions of patients with PBD reported in previous studies [35–37]. However, our patients had a normal metabolic profile, in contrast to the clinical manifestations of patients with PBDs. Patients with PBD show elevated levels of VLCFAs. Although, normal plasma VLCFAs have also been reported in a few patients [22,33,64]. The absence of the corpus callosum and widely spaced eyes (hypertelorism) have led neonatologists to misdiagnose these patients with acrocallosal syndrome. However, cleft palate, congenital heart defects, omphalocele, and dentition defects were absent in our patient.

**Figure 3.**
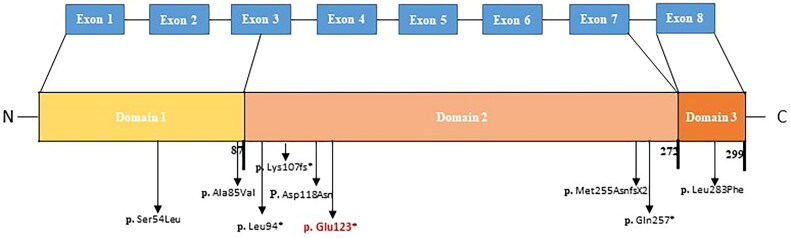
The identified mutations of PEX19 presented with respect to Pex19 domains. The mutation (p.Glu123*) identified in this study is indicated in red.

Mutations in *PEX19* have been associated with multisystem involvement, resulting in severe phenotypes, such as hypotonia, hydrocephalus, cardiac anomaly, genital abnormalities, dense bones, abnormal facial features, and early neonatal death [36]. There are very few reported cases of *PEX19* gene mutations and only eight studies have been published, of which four had missense, two nonsense, and two had frameshift mutations diagnosed with developmental disorder, ZS, PBDs, and myoclonic epilepsy (Figure 3, Table 2). Patients with missense mutations (p.Ala85Val and p.Ser54Leu) had late-onset mild clinical symptoms with long-term survival [30,31], whereas other reported *PEX19* patients presented the ZS phenotype with early death. These patients suffered severe clinical symptoms, such as hypotonia, hydrocephalus, cardiac anomaly, wide open fontanelles, facial dysmorphism, and dense bones [36,37]. One of the reported *PEX19* cases with an insertion mutation c.763_764insA showed a less severe phenotype with milder biochemical abnormalities and survived for up to 16 months after developing liver dysfunction and renal tubular defects [35]. Based on the missense mutation identified in *PEX19* gene in our patient, we hypothesized that she might develop other complications such as liver dysfunction and renal defect over time, although her metabolic profile is currently unremarkable and she has mild clinical features. Our patient is stable on nasal and OGT feeding.

PEX19 has three conserved domains: D1 (1-87 aa), D2 (88-272 aa), and D3 (273-299 aa) [68]. The C-terminal part of the Peroxin19 polypeptide has an exclusive CAAX-box that harbors the intrinsic property of farnesylation and undergoes a conformational change that increases its binding affinity for PMPs. It also has distinct binding sites for Peroxin3, one at the N-terminal part and another in domain 2 [69–71]. The identified mutation (c.367C > T; p. Gln123*) in a patient from family A is located in exon 4 of *PEX19*, which is predicted to cause premature termination of the mRNA transcript in domain 2 (Figure 3). This premature mRNA transcript has a termination codon, localized more than 50 nucleotides upstream of the exon-exon junction at the 3′ end, and is predicted to undergo nonsense-mediated mRNA decay in cells. Therefore, the patient did not have functional PEX19 polypeptide. In mammalian cells, the NMD pathway operates on all mRNA transcripts with premature termination codons (PTC) located 50–55 nucleotides upstream of the final exon-exon junction [72–74].

**Figure 4.**
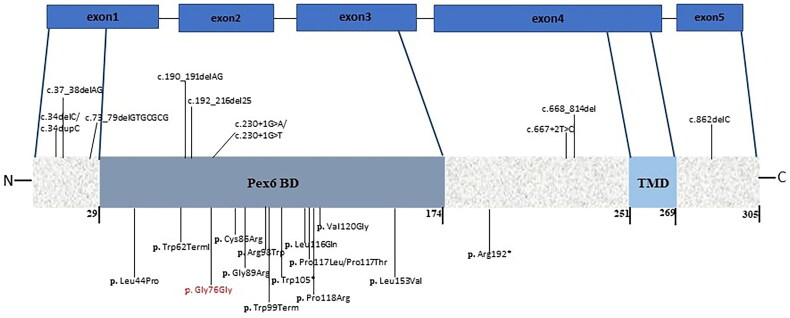
The figure highlights the Pex6 binding domain (Pex6BD) and transmembrane domain (TMD) in Pex26. The reported mutations of PEX26 involved in PBD, ZS, and MD are shown with respect to the Pex26 domains. The mutation (p.Gly76Gly) identified in this study is indicated in red.

A total of 32 pathogenic mutations in *PEX26* have been described in the literature (Figure 4). Most PBDs patients with PEX26 mutations have metabolic disorders. Nine PEX26 patients were diagnosed with ZS (Table 3) [80]. Detailed clinical phenotypes of four ZS infants with *PEX26* mutations have been studied in Saudi Arabia, Tunisia, and America [77, 81–83]. These patients had common postnatal clinical symptoms resembling those of our patient with the *PEX26* variant. The shared phenotypes include hypotonia, liver dysfunction, facial deformities, prominent forehead, wide cranial sutures, low-set ears, and enlarged fontanelles with involvement of ventricular dilation, fundus lesions, and skeletal abnormalities comprising bilateral clubfoot and chondrodysplasia. Analysis of the WES data identified a homozygous synonymous variant (c.228C > T; p.Gly76Gly) in *PEX26* in family B patients. The mutation has been found in the HGMD database and has been reported previously in a Saudi patient with intellectual disability and inborn errors of metabolism [58]. Our patient from family B had normal metabolic screening but suffered neuronal defects, such as the absence of corpus callosum and ventriculomegaly.

**Table 3.** List of all the identified mutations in *PEX26* (from HGMD professional).

HGVS	Amino Acid/Codon Change	Reported phenotype	References
c.230 + 1G > A	IVS1 ds G-A +1	Zellweger syndrome	[75]
c.230 + 1G > T	IVS1 ds G-T +1	Peroxisome biogenesis disorder	[51]
c.667 + 2T > C	IVS3 ds T-C +2	Peroxisome biogenesis disorder	[26]
c.34delC	ACCTCT^**^9^**GCAGcCCCCCTCAGG	Peroxisome biogenesis disorder	[76]
c.37_38delAG	AGCCCCC^**^12^**CTCagGGGGCTCGGG	Peroxisome biogenesis disorder	[26]
c.73_79delGTGCGCG	CAGCGAG^**^24^**CCGgtgcgcgCGGTCCCGGC	Zellweger syndrome	[33]
c.190_191delAG	GGCCTGG^**^63^**CAGagTCTGGCCAAC	Peroxisomal biogenesis disorder	[30]
c.862delC	CCAGCTC^**^287^**TTCcGCTGGATCCG	Peroxisome biogenesis disorder	[51]
c.34dupC	TCTGCA^**^10^**GCCCcCCCTCAGGGG	Zellweger syndrome	[52]
c.192_216del25	25 bp, c.192-216	Peroxisome biogenesis disorder	[26]
Not yet available	c.668_814del	Zellweger syndrome	[33]
c.131T > C	Leu44Pro	Peroxisome biogenesis disorder	[51]
c.185G > A	Trp62Term	Zellweger syndrome	[33]
c.228C > T	Gly76Gly	Intellectual disability/Inborn errors of metabolism	[58]/ Present study
c.256T > C	Cys86Arg	Zellweger syndrome	[77]
c.265G > A	Gly89Arg	Zellweger syndrome	[52]
c.292C > T	Arg98Trp	Peroxisome biogenesis disorder	[52]
c.296G > A	Trp99Term	Peroxisome biogenesis disorder	[26]
c.315G > A	Trp105Term	Zellweger syndrome	[33]
c.347T > A	Leu116Gln	Zellweger syndrome	[78]
c.350C > T	Pro117Leu	Peroxisome biogenesis disorder	[51]
c.349C > A	Pro117Thr	Peroxisome biogenesis disorder	[79]
c.353C > G	Pro118Arg	Peroxisome biogenesis disorder	[26]
c.359T > G	Val120Gly	Peroxisome biogenesis disorder	[76]
c.457C > G	Leu153Val	Peroxisome biogenesis disorder	[51]

The identified exonic variant (c.228C > T) in family B is located 3 bp upstream of the canonical splice donor site at the end of exon 2. It has been shown that apart from intronic mutations that disrupt canonical splicing locations or activate cryptic exons, changes in exonic sequences may also affect the pattern of pre-mRNA splicing, suggesting that exonic mutations may have a dual effect: 1) they either introduce a new 5′ or 3′ splice site or activate the cryptic splice site, affecting pre-mRNA processing and the deletion of an exon, or 2) exonic mutations alter exonic splicing enhancers, which may result in whole exon skipping [84]. The exonic missense, synonymous, or nonsense variants cause splicing alterations that result in multiple abnormalities in pre-mRNA splicing, manifested as the generation of two different transcripts from one mutated allele: a shorter fragment lacking the whole exon or its part and another with a proper length and modified nucleotide [85].

Recent data from numerous laboratories suggest that silent genomic variants can interfere with splicing and are frequently the source of human genetic disorders [86–89]. Exonic variants localized near canonical splice sites can affect pre-mRNA processing/splicing by interfering with splice enhancers and reducing spliceosomal recognition of canonical splice sites [90]. The impact of a variant on the splice potential of a given sequence can be predicted using computational tools. A given variant can create a candidate splice sequence if it occurred within −3 to +5 of an existing GT and/or within −22 to +1 of AG dinucleotide of the gene of interest [91]. The variant (c.228C > T) in *PEX26* identified in the present study fulfils this criterion and can be checked on this ground to confirm *in silico* pathogenicity.

In conclusion, we concluded that PBDs are heterogeneous with respect to their clinical manifestations. We reported two cases of PBD belonging to Saudi families and identified a novel nonsense mutation in *PEX19* and a previously reported synonymous variant in *PEX26* both predicted as pathogenic based on bioinformatics analysis. The study patients had a generalized clinical presentation of PBDs, with the exception of normal metabolic function and stable life expectancy in the neonatal period. The patient from family B showed a characteristic, severe ZS-like clinical phenotype.

## Data Availability

The datasets used in this study have been submitted to the European Variation Archive (EVA). The accession number is PRJEB48950 and the link to the data is https://www.ebi.ac.uk/eva/?Study-Browser&browserType=sgv.

## References

[1] Huybrechts SJ, Van Veldhoven PP, Brees C, et al. Peroxisome dynamics in cultured mammalian cells. Traffic. 2009;10(11):1722–1733. doi: 10.1111/j.1600-0854.2009.00970.x. 19719477

[2] Ferdinandusse S, Meissner T, Wanders RJ, et al. Identification of the peroxisomal β-oxidation enzymes involved in the degradation of leukotrienes. Biochem Biophys Res Commun. 2002;293(1):269–273. doi: 10.1016/S0006-291X(02)00214-0. 12054595

[3] Schrader M, Fahimi HD. Peroxisomes and oxidative stress. Biochim Biophys Acta. 2006;1763(12):1755–1766. doi: 10.1016/j.bbamcr.2006.09.006. 17034877

[4] Wanders RJ, Waterham HR. Peroxisomal disorders: the single peroxisomal enzyme deficiencies. Biochim Biophys Acta. 2006b;1763(12):1707–1720. doi: 10.1016/j.bbamcr.2006.08.010. 17055078

[5] Argyriou C, D’Agostino MD, Braverman N. Peroxisome biogenesis disorders. Transl Sci Rare Dis. 2016;1(2):111–144. doi: 10.3233/TRD-160003. 29152457 PMC5678237

[6] Mannaerts G, Van Veldhoven PP, Reddy J, et al. Functions and organization of peroxisomal beta-oxidation. Ann N Y Acad Sci. 1996;804(1):99–115. doi: 10.1111/j.1749-6632.1996.tb18611.x. 8993539

[7] Waterham HR, Ebberink MS. Genetics and molecular basis of human peroxisome biogenesis disorders. Biochim Biophys Acta. 2012;1822(9):1430–1441. doi: 10.1016/j.bbadis.2012.04.006. 22871920

[8] Agrawal G, Subramani S. De novo peroxisome bio­genesis: evolving concepts and conundrums. Biochim Biophys Acta. 2016;1863(5):892–901. doi: 10.1016/j.bbamcr.2015.09.014. 26381541 PMC4791208

[9] Schrader M, Fahimi HD. The peroxisome: still a mysterious organelle. Histochem Cell Biol. 2008;129(4):421–440. doi: 10.1007/s00418-008-0396-9. 18274771 PMC2668598

[10] Tabak HF, Braakman I, van der Zand A. Peroxisome formation and maintenance are dependent on the endoplasmic reticulum. Annu Rev Biochem. 2013;82(1):723–744. doi: 10.1146/annurev-biochem-081111-125123. 23414306

[11] Wanders RJ, Waterham HR. Biochemistry of mammalian peroxisomes revisited. Annu Rev Biochem. 2006a;75(1):295–332. doi: 10.1146/annurev.biochem.74.082803.133329. 16756494

[12] Schlüter A, Fourcade S, Domènech-Estévez E, et al. PeroxisomeDB: a database for the peroxisomal proteome, functional genomics and disease. Nucleic Acids Res. 2007;35(Database issue):D815–D822. doi: 10.1093/nar/gkl935. 17135190 PMC1747181

[13] Ferdinandusse S, Falkenberg KD, Koster J, et al. ACBD5 deficiency causes a defect in peroxisomal very long-chain fatty acid metabolism. J Med Genet. 2017;54(5):330–337. doi: 10.1136/jmedgenet-2016-104132. 27799409

[14] Mosser J, Douar A-M, Sarde C-O, et al. Putative X-linked adrenoleukodystrophy gene shares unexpected homology with ABC transporters. Nature. 1993;361(6414):726–730. doi: 10.1038/361726a0. 8441467

[15] Waterham HR, Ferdinandusse S, Wanders RJ. Human disorders of peroxisome metabolism and biogenesis. Biochim Biophys Acta. 2016;1863(5):922–933. doi: 10.1016/j.bbamcr.2015.11.015. 26611709

[16] Honsho M, Okumoto K, Tamura S, et al. Peroxisome biogenesis disorders. Adv Exp Med Biol. 2020;1299:45–54. doi: 10.1007/978-3-030-60204-8_4. 33417206

[17] Kashgari A. Neonate with classic Zellweger syndrome. Int J Pediatr Adolesc Med. 2019;6(4):165–166. doi: 10.1016/j.ijpam.2019.11.002. 31890844 PMC6926218

[18] Rosewich H, Waterham H, Poll-The BT, et al. Clinical utility gene card for: zellweger syndrome spectrum. Eur J Hum Genet. 2015;23(8):1111–1111. doi: 10.1038/ejhg.2014.250. PMC4795113 25407003

[19] Berendse K, Engelen M, Ferdinandusse S, et al. Zellweger spectrum disorders: clinical manifestations in patients surviving into adulthood. J Inherit Metab Dis. 2016;39(1):93–106. doi: 10.1007/s10545-015-9880-2. 26287655 PMC4710674

[20] Oswald G, Lawson C, Raymond G, et al. Rhizomelic chondrodysplasia punctata type 1 and fulminant neonatal respiratory failure, a case report and discussion of pathophysiology. Am J Med Genet A. 2011;155A(12):3160–3163. doi: 10.1002/ajmg.a.34331. 22052861

[21] Raha BK, Haque Z, Jahan N, et al. Rhizomelic Chondrodysplasia Punctata (RCDP) in a Newborn. J Bangladesh Coll Phys. 2015;32(3):174–177. doi: 10.3329/jbcps.v32i3.26058.

[22] Braverman NE, Raymond GV, Rizzo WB, et al. Peroxisome biogenesis disorders in the Zellweger spectrum: an overview of current diagnosis, clinical manifestations, and treatment guidelines. Mol Genet Metab. 2016;117(3):313–321. doi: 10.1016/j.ymgme.2015.12.009. 26750748 PMC5214431

[23] Steinberg S, Raymond G, Braverman N, et al. Zellweger spectrum disorder. GeneReviews. Seattle, WA University of Washington; 2017. 20301621

[24] Krause C, Rosewich H, Thanos M, et al. Identification of novel mutations in PEX2, PEX6, PEX10, PEX12, and PEX13 in Zellweger spectrum patients. Hum Mutat. 2006;27(11):1157–1157. doi: 10.1002/humu.9462. 17041890

[25] Shimozawa N, Nagase T, Takemoto Y, et al. Genetic heterogeneity of peroxisome biogenesis disorders among Japanese patients: evidence for a founder haplotype for the most common PEX10 gene mutation. Am J Med Genet A. 2003;120A(1):40–43. doi: 10.1002/ajmg.a.20030. 12794690

[26] Steinberg S, Chen L, Wei L, et al. The PEX Gene Screen: molecular diagnosis of peroxisome biogenesis disorders in the Zellweger syndrome spectrum. Mol Genet Metab. 2004;83(3):252–263. doi: 10.1016/j.ymgme.2004.08.008. 15542397

[27] Berger J, Dorninger F, Forss-Petter S, et al. Peroxisomes in brain development and function. Biochim Biophys Acta. 2016;1863(5):934–955. doi: 10.1016/j.bbamcr.2015.12.005. 26686055 PMC4880039

[28] Braverman NE, D’Agostino MD, MacLean GE. Peroxisome biogenesis disorders: biological, clinical and pathophysiological perspectives. Dev Disabil Res Rev. 2013;17(3):187–196. doi: 10.1002/ddrr.1113. 23798008

[29] Ebberink MS, Koster J, Visser G, et al. A novel defect of peroxisome division due to a homozygous non-sense mutation in the PEX11β gene. J Med Genet. 2012;49(5):307–313. doi: 10.1136/jmedgenet-2012-100778. 22581968

[30] Alshenaifi J, Ewida N, Anazi S, et al. The many faces of peroxisomal disorders: lessons from a large Arab cohort. Clin Genet. 2019;95(2):310–319. doi: 10.1111/cge.13481. 30561787

[31] Courage C, Oliver KL, Park EJ, et al. Progressive myoclonus epilepsies—Residual unsolved cases have marked genetic heterogeneity including dolichol-dependent protein glycosylation pathway genes. Am J Hum Genet. 2021;108(4):722–738. doi: 10.1016/j.ajhg.2021.03.013. 33798445 PMC8059372

[32] Turner TN, Wilfert AB, Bakken TE, et al. Sex-based analysis of de novo variants in neurodevelopmental disorders. Am J Hum Genet. 2019;105(6):1274–1285. doi: 10.1016/j.ajhg.2019.11.003. 31785789 PMC6904808

[33] Ebberink MS, Mooijer PA, Gootjes J, et al. Genetic classification and mutational spectrum of more than 600 patients with a Zellweger syndrome spectrum disorder. Hum Mutat. 2011;32(1):59–69. doi: 10.1002/humu.21388. 21031596

[34] Deciphering Developmental Disorders Study. Prevalence and architecture of de novo mutations in developmental disorders. Nature. 2017;542(7642):433–438. doi:10.1038/nature21062 PMC6016744 28135719

[35] Mohamed S, El‐Meleagy E, Nasr A, et al. A mutation in PEX19 causes a severe clinical phenotype in a patient with peroxisomal biogenesis disorder. Am J Med Genet A. 2010;152A(9):2318–2321. doi: 10.1002/ajmg.a.33560. 20683989

[36] Matsuzono Y, Kinoshita N, Tamura S, et al. Human PEX19: cDNA cloning by functional complementation, mutation analysis in a patient with Zellweger syndrome, and potential role in peroxisomal membrane assembly. Proc Natl Acad Sci U S A. 1999;96(5):2116–2121. doi: 10.1073/pnas.96.5.2116. 10051604 PMC26746

[37] Adiyapatham S, Murugesan A. Novel mutation causing Zellweger syndrome. BMJ Case Rep. 2023;16(3):e252014. doi: 10.1136/bcr-2022-252014. PMC10030475 36931687

[38] Honsho M, Tamura S, Shimozawa N, et al. Mutation in PEX16 is causal in the peroxisome-deficient Zellweger syndrome of complementation group D. Am J Hum Genet. 1998;63(6):1622–1630. doi: 10.1086/302161. 9837814 PMC1377633

[39] Muntau AC, Mayerhofer PU, Paton BC, et al. Defective peroxisome membrane synthesis due to mutations in human PEX3 causes Zellweger syndrome, complementation group G. Am J Hum Genet. 2000;67(4):967–975. doi: 10.1086/303071. 10958759 PMC1287898

[40] Shimozawa N, Suzuki Y, Zhang Z, et al. Identification of PEX3 as the gene mutated in a Zellweger syndrome patient lacking peroxisomal remnant structures. Hum Mol Genet. 2000;9(13):1995–1999. doi: 10.1093/hmg/9.13.1995. 10942428

[41] Fujiki Y. Peroxisome biogenesis and peroxisome biogenesis disorders. FEBS Lett. 2000a;476(1-2):42–46. doi: 10.1016/s0014-5793(00)01667-7. 10878247

[42] Weller S, Gould SJ, Valle D. Peroxisome biogenesis disorders. Annu Rev Genomics Hum Genet. 2003;4(1):165–211. doi: 10.1146/annurev.genom.4.070802.110424. 14527301

[43] Kammerer S, Arnold N, Gutensohn W, et al. Genomic organization and molecular characterization of a gene encoding HsPXF, a human peroxisomal farnesylated protein. Genomics. 1997;45(1):200–210. doi: 10.1006/geno.1997.4914. 9339377

[44] Götte K, Girzalsky W, Linkert M, et al. Pex19p, a farnesylated protein essential for peroxisome biogenesis. Mol Cell Biol. 1998;18(1):616–628. doi: 10.1128/MCB.18.1.616. 9418908 PMC121529

[45] Rucktäschel R, Thoms S, Sidorovitch V, et al. Farnesylation of pex19p is required for its structural integrity and function in peroxisome biogenesis. J Biol Chem. 2009;284(31):20885–20896. doi: 10.1074/jbc.M109.016584. 19451657 PMC2742854

[46] Giannopoulou E-A, Emmanouilidis L, Sattler M, et al. Towards the molecular mechanism of the integration of peroxisomal membrane proteins. Biochim Biophys Acta. 2016;1863(5):863–869. doi: 10.1016/j.bbamcr.2015.09.031. 26434995 PMC4819957

[47] Sacksteder KA, Jones JM, South ST, et al. PEX19 binds multiple peroxisomal membrane proteins, is predominantly cytoplasmic, and is required for peroxisome membrane synthesis. J Cell Biol. 2000;148(5):931–944. doi: 10.1083/jcb.148.5.931. 10704444 PMC2174547

[48] Sato Y, Shibata H, Nakatsu T, et al. Structural basis for docking of peroxisomal membrane protein carrier Pex19p onto its receptor Pex3p. Embo J. 2010;29(24):4083–4093. doi: 10.1038/emboj.2010.293. 21102411 PMC3018794

[49] Fransen M, Lismont C. Redox signaling from and to peroxisomes: progress, challenges, and prospects. Antioxid Redox Signal. 2019;30(1):95–112. doi: 10.1089/ars.2018.7515. 29433327

[50] Lismont C, Revenco I, Fransen M. Peroxisomal hydrogen peroxide metabolism and signaling in health and disease. Int J Mol Sci. 2019;20(15):3673. doi: 10.3390/ijms20153673. 31357514 PMC6695606

[51] Furuki S, Tamura S, Matsumoto N, et al. Mutations in the peroxin Pex26p responsible for peroxisome biogenesis disorders of complementation group 8 impair its stability, peroxisomal localization, and interaction with the Pex1p· Pex6p complex. J Biol Chem. 2006;281(3):1317–1323. doi: 10.1074/jbc.M510044200. 16257970

[52] Matsumoto N, Tamura S, Furuki S, et al. Mutations in novel peroxin gene PEX26 that cause peroxisome-biogenesis disorders of complementation group 8 provide a genotype-phenotype correlation. Am J Hum Genet. 2003;73(2):233–246. doi: 10.1086/377004. 12851857 PMC1180364

[53] Nazarko TY. Pexophagy is responsible for 65% of cases of peroxisome biogenesis disorders. Autophagy. 2017;13(5):991–994. doi: 10.1080/15548627.2017.1291480. 28318378 PMC5446054

[54] Weller S, Cajigas I, Morrell J, et al. Alternative splicing suggests extended function of PEX26 in peroxisome biogenesis. Am J Hum Genet. 2005;76(6):987–1007. doi: 10.1086/430637. 15858711 PMC1196456

[55] Zhang R, Chen L, Jiralerspong S, et al. Recovery of PEX1-Gly843Asp peroxisome dysfunction by small-molecule compounds. Proc Natl Acad Sci U S A. 2010;107(12):5569–5574. doi: 10.1073/pnas.0914960107. 20212125 PMC2851769

[56] Fujiki Y, Abe Y, Imoto Y, et al. Recent insights into peroxisome biogenesis and associated diseases. J Cell Sci. 2020b;133(9):jcs236943. doi: 10.1242/jcs.236943. 32393673

[57] Richards S, Aziz N, Bale S, et al. Standards and guidelines for the interpretation of sequence variants: a joint consensus recommendation of the American College of Medical Genetics and Genomics and the Association for Molecular Pathology. Genet Med. 2015;17(5):405–424. doi: 10.1038/gim.2015.30. 25741868 PMC4544753

[58] Abouelhoda M, Sobahy T, El-Kalioby M, et al. Clinical genomics can facilitate countrywide estimation of autosomal recessive disease burden. Genet Med. 2016;18(12):1244–1249. doi: 10.1038/gim.2016.37. 27124789

[59] Stradomska TJ. Peroxisomal disorders. Postepy Biochem. 2018;64(4):359–367. doi: 10.18388/pb.2018_150. 30656921

[60] Thoms S, Grønborg S, Gärtner J. Organelle interplay in peroxisomal disorders. Trends Mol Med. 2009;15(7):293–302. doi: 10.1016/j.molmed.2009.05.002. 19560974

[61] Van Veldhoven PP. Biochemistry and genetics of inherited disorders of peroxisomal fatty acid metabolism [S]. J Lipid Res. 2010;51(10):2863–2895. doi: 10.1194/jlr.R005959. 20558530 PMC2936746

[62] Wanders RJ. Metabolic functions of peroxisomes in health and disease. Biochimie. 2014;98:36–44. doi: 10.1016/j.biochi.2013.08.022. 24012550

[63] Fidaleo M. Peroxisomes and peroxisomal disorders: the main facts. Exp Toxicol Pathol. 2010;62(6):615–625. doi: 10.1016/j.etp.2009.08.008. 19740638

[64] Klouwer FC, Berendse K, Ferdinandusse S, et al. Zellweger spectrum disorders: clinical overview and management approach. Orphanet J Rare Dis. 2015;10(1):151. doi: 10.1186/s13023-015-0368-9. 26627182 PMC4666198

[65] Motley A, Tabak H, Smeitink J, et al. Non-rhizomelic and rhizomelic chondrodysplasia punctata within a single complementation group. Biochim Biophys Acta. 1996;1315(3):153–158. doi: 10.1016/0925-4439(95)00114-x. 8611652

[66] Wangler MF, Hubert L, Donti TR, et al. A metabolomic map of Zellweger spectrum disorders reveals novel disease biomarkers. Genet Med. 2018;20(10):1274–1283. doi: 10.1038/gim.2017.262. 29419819 PMC7605708

[67] Kheir AE. Zellweger syndrome: a cause of neonatal hypotonia and seizures. Sudan J Paediatr. 2011;11(2):54–58. 27493320 PMC4949836

[68] Mayerhofer PU, Kattenfeld T, Roscher AA, et al. Two splice variants of human PEX19 exhibit distinct functions in peroxisomal assembly. Biochem Biophys Res Commun. 2002;291(5):1180–1186. doi: 10.1006/bbrc.2002.6568. 11883941

[69] Fransen M, Wylin T, Brees C, et al. Human pex19p binds peroxisomal integral membrane proteins at regions distinct from their sorting sequences. Mol Cell Biol. 2001;21(13):4413–4424. doi: 10.1128/MCB.21.13.4413-4424.2001. 11390669 PMC87101

[70] Snyder WB, Faber KN, Wenzel TJ, et al. Pex19p interacts with Pex3p and Pex10p and is essential for peroxisome biogenesis in Pichia pastoris. Mol Biol Cell. 1999a;10(6):1745–1761. doi: 10.1091/mbc.10.6.1745. 10359594 PMC25367

[71] Snyder WB, Koller A, Choy AJ, et al. Pex17p is required for import of both peroxisome membrane and lumenal proteins and interacts with Pex19p and the peroxisome targeting signal–receptor docking complex in Pichia pastoris. Mol Biol Cell. 1999b;10(12):4005–4019. doi: 10.1091/mbc.10.12.4005. 10588639 PMC25739

[72] Nagy E, Maquat LE. A rule for termination-codon position within intron-containing genes: when nonsense affects RNA abundance. Trends Biochem Sci. 1998;23(6):198–199. doi: 10.1016/s0968-0004(98)01208-0. 9644970

[73] Zhang J, Sun X, Qian Y, et al. At least one intron is required for the nonsense-mediated decay of triosephosphate isomerase mRNA: a possible link between nuclear splicing and cytoplasmic translation. Mol Cell Biol. 1998a;18(9):5272–5283. doi: 10.1128/MCB.18.9.5272. 9710612 PMC109113

[74] Zhang J, Sun X, Qian Y, et al. Intron function in the nonsense-mediated decay of β-globin mRNA: indications that pre-mRNA splicing in the nucleus can influence mRNA translation in the cytoplasm. RNA. 1998b;4(7):801–815. doi: 10.1017/s1355838298971849. 9671053 PMC1369660

[75] Shen O, Michaelson‐Cohen R, Gross‐Tsur V, et al. Prenatal observation of nystagmus, cataracts, and brain abnormalities in a case of Zellweger spectrum disorder syndrome. Prenat Diagn. 2016;36(9):894–895. doi: 10.1002/pd.4872. 27392320

[76] Hong S, Wang L, Zhao D, et al. Clinical utility in infants with suspected monogenic conditions through next‐generation sequencing. Mol Genet Genomic Med. 2019;7(6):e684. doi: 10.1002/mgg3.684. 30968598 PMC6565546

[77] Stowe RC, Agarwal S. Novel PEX26 mutation causing Zellweger syndrome presenting as feeding intolerance and hypotonia. Pediatr Neurol. 2017;75:96–97. doi: 10.1016/j.pediatrneurol.2017.06.012. 28823628

[78] Semenova NA, Kurkina MV, Marakhonov AV, et al. A novel mutation in the PEX26 gene in a family from Dagestan with members affected by Zellweger spectrum disorder. Mol Genet Metab Rep. 2021;27:100754. doi: 10.1016/j.ymgmr.2021.100754. 33912394 PMC8065337

[79] Bahena P, Daftarian N, Maroofian R, et al. Unraveling the genetic complexities of combined retinal dystrophy and hearing impairment. Hum Genet. 2022;141(3-4):785–803. doi: 10.1007/s00439-021-02303-1. 34148116 PMC9035000

[80] He Y, Lin SB, Li W-X, et al. PEX26 gene genotype-phenotype correlation in neonates with Zellweger syndrome. Transl Pediatr. 2021;10(7):1825–1833. doi: 10.21037/tp-21-103. 34430430 PMC8349955

[81] Al-Sayed M, Al-Hassan S, Rashed M, et al. Preimplantation genetic diagnosis for Zellweger syndrome. Fertil Steril. 2007;87(6):1468.e1461–1468.e1463. doi: 10.1016/j.fertnstert.2006.09.014. 17336976

[82] Rife E, Dunbar AE, Nelson SL, et al. Stippled chondral calcifications of the patella in Zellweger syndrome. J Pediatr. 2018;192:265. doi: 10.1016/j.jpeds.2017.09.064. 29246349

[83] Turkia B, Yangui M, Azzouz H, et al. A novel mutation in PEX 26 gene in Zellweger syndrome: a case report. Tunis Med. 2011;89(3):288–291. 21387236

[84] Wimmer K, Roca X, Beiglböck H, et al. Extensive in silico analysis of NF1 splicing defects uncovers determinants for splicing outcome upon 5′ splice‐site disruption. Hum Mutat. 2007;28(6):599–612. doi: 10.1002/humu.20493. 17311297

[85] Nissim-Rafinia M, Kerem B. Splicing regulation as a potential genetic modifier. Trends Genet. 2002;18(3):123–127. doi: 10.1016/s0168-9525(01)02619-1. 11858835

[86] Cartegni L, Chew SL, Krainer AR. Listening to silence and understanding nonsense: exonic mutations that affect splicing. Nat Rev Genet. 2002;3(4):285–298. doi: 10.1038/nrg775. 11967553

[87] Fernandez-Cadenas I, Andreu A, Gamez J, et al. Splicing mosaic of the myophosphorylase gene due to a silent mutation in McArdle disease. Neurology. 2003;61(10):1432–1434. doi: 10.1212/wnl.61.10.1432. 14638972

[88] Pagani F, Stuani C, Tzetis M, et al. New type of disease causing mutations: the example of the composite exonic regulatory elements of splicing in CFTR exon 12. Hum Mol Genet. 2003;12(10):1111–1120. doi: 10.1093/hmg/ddg131. 12719375

[89] Thude H, Hundrieser J, Wonigeit K, et al. A point mutation in the human CD45 gene associated with defective splicing of exon A. Eur J Immunol. 1995;25(7):2101–2106. doi: 10.1002/eji.1830250745. 7621884

[90] Soukarieh O, Gaildrat P, Hamieh M, et al. Exonic splicing mutations are more prevalent than currently estimated and can be predicted by using in silico tools. PLoS Genet. 2016;12(1):e1005756. doi: 10.1371/journal.pgen.1005756. 26761715 PMC4711968

[91] Pagani F, Baralle FE. Genomic variants in exons and introns: identifying the splicing spoilers. Nat Rev Genet. 2004;5(5):389–396. doi: 10.1038/nrg1327. 15168696

[92] Gould SJ, Valle D. Peroxisome biogenesis disorders: genetics and cell biology. Trends Genet. 2000;16(8):340–345. doi: 10.1016/s0168-9525(00)02056-4. 10904262

